# Malaria disrupts the rhesus macaque gut microbiome

**DOI:** 10.3389/fcimb.2022.1058926

**Published:** 2023-01-13

**Authors:** Danielle N. Farinella, Sukhpreet Kaur, ViLinh Tran, Monica Cabrera-Mora, Chester J. Joyner, Stacey A. Lapp, Suman B. Pakala, Mustafa V. Nural, Jeremy D. DeBarry, Jessica C. Kissinger, Dean P. Jones, Alberto Moreno, Mary R. Galinski, Regina Joice Cordy

**Affiliations:** ^1^ Department of Biology, Wake Forest University, Winston-Salem, NC, United States; ^2^ Division of Pulmonary, Allergy, Critical Care, and Sleep Medicine, Department of Medicine, School of Medicine, Emory University, Atlanta, GA, United States; ^3^ Yerkes National Primate Research Center, Emory University, Atlanta, GA, United States; ^4^ Department of Infectious Diseases, University of Georgia, Athens, GA, United States; ^5^ Institute of Bioinformatics, University of Georgia, Athens, GA, United States; ^6^ Center for Tropical and Emerging Global Diseases, University of Georgia, Athens, GA, United States; ^7^ Emory Vaccine Center, Emory University, Atlanta, GA, United States; ^8^ Department of Genetics, University of Georgia, Athens, GA, United States; ^9^ Division of Infectious Diseases, Department of Medicine, Emory University, Atlanta, GA, United States

**Keywords:** microbiome, malaria, primate, relapse, *Plasmodium*, metabolomics

## Abstract

Previous studies have suggested that a relationship exists between severity and transmissibility of malaria and variations in the gut microbiome, yet only limited information exists on the temporal dynamics of the gut microbial community during a malarial infection. Here, using a rhesus macaque model of relapsing malaria, we investigate how malaria affects the gut microbiome. In this study, we performed 16S sequencing on DNA isolated from rectal swabs of rhesus macaques over the course of an experimental malarial infection with *Plasmodium cynomolgi* and analyzed gut bacterial taxa abundance across primary and relapsing infections. We also performed metabolomics on blood plasma from the animals at the same timepoints and investigated changes in metabolic pathways over time. Members of Proteobacteria (family *Helicobacteraceae*) increased dramatically in relative abundance in the animal’s gut microbiome during peak infection while Firmicutes (family *Lactobacillaceae* and *Ruminococcaceae*), Bacteroidetes (family *Prevotellaceae*) and Spirochaetes amongst others decreased compared to baseline levels. Alpha diversity metrics indicated decreased microbiome diversity at the peak of parasitemia, followed by restoration of diversity post-treatment. Comparison with healthy subjects suggested that the rectal microbiome during acute malaria is enriched with commensal bacteria typically found in the healthy animal’s mucosa. Significant changes in the tryptophan-kynurenine immunomodulatory pathway were detected at peak infection with P. *cynomolgi*, a finding that has been described previously in the context of P. *vivax* infections in humans. During relapses, which have been shown to be associated with less inflammation and clinical severity, we observed minimal disruption to the gut microbiome, despite parasites being present. Altogether, these data suggest that the metabolic shift occurring during acute infection is associated with a concomitant shift in the gut microbiome, which is reversed post-treatment.

## 1 Introduction

Infection by *Plasmodium* species persists as a global health issue, resulting in over 200 million cases of malaria and approximately 600,000 deaths annually (World Health Organization (2021)). The malarial parasite has coevolved with its insect and vertebrate hosts over tens of thousands of years and causes infections ranging from mild/asymptomatic to severe (Ewald (1983); Waters et al. (1991); Mackintosh et al. (2004)). Subclinical infections in partially immune individuals, especially in regions of high malaria endemicity contribute to sustained transmission (Ippolito et al. (2018)). Gut permeability is closely linked to intestinal microbiota and elements of the mucosal immune system. A balanced intestinal microbiome not only helps maintain the microbial homeostasis and immunologic tolerance but also helps modulate the metabolic process that influences the intestinal permeability (Lim et al. (2018); Zheng et al. (2020)). This can occur due to effects on the production of short chain fatty acids that play an important role in enterocyte development or through bacterial factors that influence intestinal barrier function.

The host-parasite relationship during malarial infections prominently manifests itself as an immune response from the host. Such deviation from immune homeostasis may directly or indirectly impact the host microbiota composition during malarial infections (Mukherjee et al. (2020)). This deviation leads to speculation that a tripartite interaction exists between host-Plasmodium-microbiota that may impact the outcome of malarial infections (Ippolito et al. (2018); Mukherjee et al. (2020)). Major advances in biomedical sequencing technologies have enabled research on elucidating the role of the gut microbiome in malarial pathophysiology. A considerable number of studies have investigated the impact of the gut microbiota of mosquitoes on malarial transmission through interference with *Plasmodium* colonization in the gut, and, by affecting different aspects of mosquito physiology, notably impacting the mosquito lifespan (Boissiere et al. (2012); Romoli and Gendrin (2018); Zoure et al. (2020)). However, there are limited reports on the potential changes in the gut microbiota of mammalian hosts upon acute malarial infection and relapse episodes.

Recent studies support that gut microbiota modulates *Plasmodium* infections in mammals (Yooseph et al. (2015); Villarino et al. (2016); Mukherjee et al. (2020)). In a murine model, anti-*α* -gal antibodies induced by gut pathobiont *Escherichia coli* O86:B7 have been shown to be cytotoxic to *Plasmodium* sporozoites, thus protecting the mice from mosquito-transmitted *Plasmodium* infection (Yilmaz et al. (2014)). Early work suggests bidirectional associations between malaria and the mammalian microbiome relating to disease phenotype, infection risk and intestinal dysbiosis (Ippolito et al. (2018)). In 2015, a 16S rRNA analysis by Mooney at al. demonstrated dysbiosis in mouse gut microbiota upon infection by *Plasmodium yoelii* that was linked to clinical disease outcomes. They observed a reduction in the Firmicutes/Bacteroidetes ratio and Proteobacteria abundance in the gut of the mice which led to a decrease in the resistance to intestinal colonization with non-typhoidal Salmonella during a concurrent malaria parasite infection (Mooney et al. (2015)). In another 16S rRNA analysis, *Plasmodium berghei* ANKA infection was linked with increased Proteobacteria and reduced Firmicutes in gut microbiomes of mice. The authors indicated that altered gut microbiome profiles upon malarial infection in mice were associated with intestinal pathological changes including detachment of epithelia in large intestines and increased intestinal permeability (Taniguchi et al. (2015)).

Studies have suggested that alterations in the mammalian host gut microbiota may influence immune responses of the host and clinical outcomes of malarial infections. In the rodent-P. yoelii malaria model system, mice acquired from different vendors, with different gut microbiota, exhibited striking differences in disease severity. These differences were linked to a particular composition of gut microbiota with increased abundance of *Lactobacillus* and *Bifidobacterium* spp. in resistant mice that exhibited elevated humoral responses compared to susceptible mice (Villarino et al. (2016)). Further, in humans, an increased abundance of the bacterial taxa *Bifidobacterium* and Streptococcus in the gut was related to a lower risk of P. *falciparum* infection (Yooseph et al. (2015)).

While host microbiota may impact malarial disease progression, *Plasmodium* infection and disease may also impact host microbiota function and abundance. During *Plasmodium* infection, host immune responses, such as increases in IFN-*γ* (Yang et al. (2014)) and TNF-*α*, can lead to decreases in occludin expression and other tight junction proteins, causing increased membrane permeability (Martini et al. (2017)). This disruption may cause selective pressures due to shifts in nutrients, or due to an introduction of new taxa to the lumen, leading to changes in gut microbiota abundance, and thus changes in gut microbiome function.

Changes in microbiota abundance and function can also result in changes in gut and bloodstream metabolite abundance. Tryptophan, an essential amino acid, is known to decrease in the bloodstream during *Plasmodium* infections and has been previously associated with disease severity (Leopold et al. (2019)). Gut microbes can degrade tryptophan to produce compounds such as indole, indole propionic acid, and indole acetic acid. Tryptophan is also degraded by the host to produce quinolinate (via the kynurenine pathway) and serotonin (Bansal et al. (2010); Liu et al. (2019)). Several of these metabolites are known to affect gut barrier integrity. For example, indole has been shown to improve intestinal barrier integrity by inducing the expression of claudin protein-encoding genes, which increases the expression of the tight junction proteins TJP1, TJP3, and TJP4 downstream (Bansal et al. (2010)).

Most tryptophan in the body is degraded *via* the host’s kynurenine pathway, in which the tryptophan to kynurenine reaction is catalyzed by heme-containing enzymes tryptophan 2,3-dioxygenase (TDO) or indoleamine-2,3-dioxygenase (IDO). Prior research has found that when IDO expression is increased in the gut mucosal layer, gastric inflammation is decreased, leading to microbial persistence of IDO expressive organisms in the mucosal layer (Larussa et al. (2015)). Kynurenine is a powerful immune regulator, increasing T-cell apoptosis and the conversion of helper T-cells to anti-inflammatory T-cells (Boros and Vecsei, 2019; Rothhammer and Quintana, 2019).

Here, based on rectal swab data, we describe dysbiosis of the host gut microbiome in a *Plasmodium cynomolgi*-rhesus macaque model of relapsing malaria. We present data on the changes in the bacterial community structure and function upon P. *cynomolgi* infection of malaria-naïve rhesus macaques, from baseline to the first peak of parasitemia, and in comparison with clinically mild relapses (Joyner et al. (2019)); that is, subsequent relapsing parasitemia that developed as a result of the activation of hypnozoites in the liver (Imwong et al. (2019)). We also compared the microbiome composition with published data on healthy rhesus macaques (Yasuda et al. (2016)). Finally, we profile the temporal dynamics of metabolites in the bloodstream of these same animals during P. *cynomolgi* infection to gain a better understanding of the host metabolic condition during the course of these infections.

## 2 Materials and methods

### 2.1 Rhesus macaque study model and parasite infections

**Figure 1:**
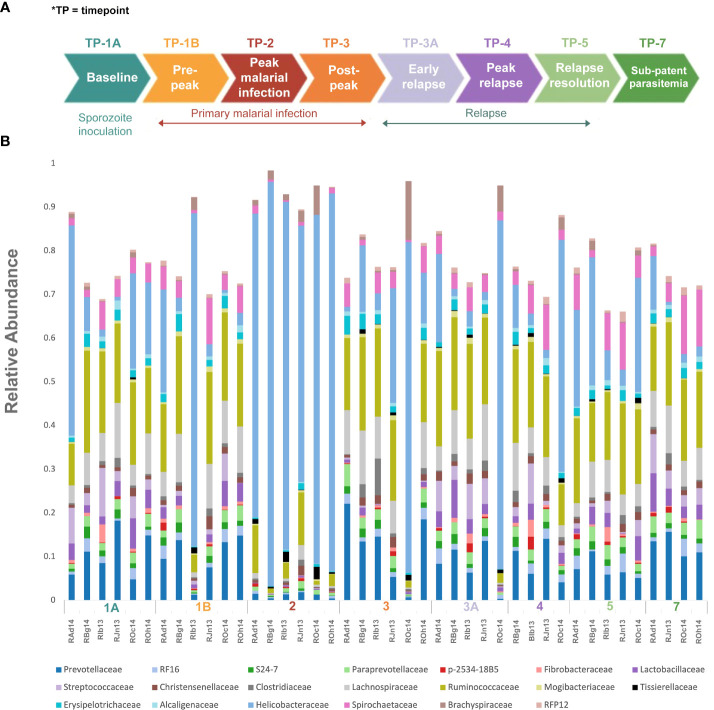
Experimental design and gut microbiota composition of rhesus macaques during a longitudinal P. *cynomolgi* infection study. **(A)** Experimental schematic for the longitudinal study of P. *cynomolgi* infection in rhesus macaques. Baseline TP1A for each monkey before inoculation of P. *cynomolgi* M/B sporozoites serves as comparison for the rest of the experiment. Patent parasitemic period TP1B represents progressive increase of infected RBCs in the blood followed by peak of infection at TP2. At post-peak TP3, a decrease in hemoglobin levels, and onset of anemia may be an indication of decreased RBC production or excessive destruction of uninfected RBCs (hemolysis). TP3A is a patent parasitemic period with increase in infected RBCs in the blood followed by a progressive increase by TP4. TP5 is indicative of subpatent parasitemia with samples representative of the period between relapses. Subpatent parasitemia at TP7 is below the microscopic threshold. **(B)** Relative abundance of predominant bacterial families in the gut of rhesus macaques (n=6) during this longitudinal study. The reference database used was Greengenes.

Rectal swabs from six rhesus macaques (n=6) were analyzed at baseline and longitudinally while infected with P. *cynomolgi*. The monkeys assigned to the study were malaria-naive (animal codes RAd14, RBg14, RIb13, RJn13, ROc14, and ROh14), of Indian origin, male, 7-13 kg and 5-6 years of age. The overall experimental design, P. *cynomolgi* M/B strain sporozoite inoculations, clinical monitoring, collection of samples, and treatment regimens have been described previously (Joyner et al. (2019); DeBarry et al. (2022)) and the scheme is depicted here in **Figure 1A**. Rectal swabs were also obtained once from four malaria-naive control rhesus macaques to perform whole metagenomics shotgun sequencing.

### 2.2 Stool sample collection and DNA extraction

Rectal swabs/feces were collected at predefined timepoints (TPs), chosen based on the infection dynamic (baseline, patent parasitemia, peak infection, post peak, relapse, and sub-patent phases) as shown in **Figure 1A** (Joyner et al. (2019)). Genomic DNA was extracted from the samples using the Power Soil DNA isolation kit (MoBio Laboratories Inc., Carlsbad, CA) according to the manufacturer’s protocols, and stored at -80°C for further downstream processing.

### 2.3 Microbiome library preparations and 16S rRNA gene sequencing

Bacterial 16S rRNA genes were amplified using bacteria-specific PCR primers for the hypervariable V3-V4 region (Klindworth et al. (2013)). The amplifications, barcoding, and sequencing experiments were performed following established protocols (Caporaso et al. (2012); Kozich et al. (2013)). Sequencing was performed on the Illumina MiSeq platform at the Yerkes Genomics Core at Emory University, resulting in 2 x 275 bp paired-end reads.

### 2.4 Bioinformatics analysis of 16S rRNA data

Quality filtering of reads and overall sequence analysis from 42 samples (six subjects at eight TPs, **Table 1**) were performed using the Quantitative Insights into Microbial Ecology, QIIME 2 software (version 2019.4). Fastq files were merged, de-multiplexed and the sequence depth for each sample was quantified (Bolyen et al. (2019)). Defined Operational Taxonomic units (OTU’s) were picked for taxonomic identity assignment using the Greengenes reference database (version 13.8) at 97% similarity. The samples were then subjected to QIIME’s core diversity analysis at 20,387 rarefaction depth for beta diversity analysis and TP comparisons. Alpha diversity indexes and their comparisons were computed in R, using the Phyloseq package (McMurdie and Holmes (2013)). The abundance of 16S reads was first normalized to proportional abundances and then the average proportional abundance for each phyla was calculated from each of the TPs (**Table S1**). The relative percentage abundances of taxa at the family level for each of the subjects, at various TPs during the *P. cynomolgi* infections, were plotted in R (**Figure 1B**). Alpha diversity was calculated within qiime2 using estimates standard in the field: Chao-1 Richness, Shannon, and Simpson indices (Finotello et al. (2018)). One-way repeated measures analysis of variance (ANOVA) was used to determine whether there were any statistically significant differences between the mean relative abundances of bacterial families across the rhesus macaque subjects at various time points. In order to identify which taxa significantly increased or decreased in abundance between timepoints, a linear discriminant analysis effect size (LEfSe) analyses was utilized with default criteria (p <0.05 by Kruskal-Wallis test; linear discriminant analysis (LDA) score >2) and subject pairing and plotted in cladograms based on phylogenetic relationship (Segata et al. (2011)). Each TP was compared to its preceding TP for LEfSe analysis. For comparisons with previously published studies, raw sequencing data were obtained from publicly available sources. Experimental data and reference sequences from Yasuda and colleagues (Yasuda et al. (2016)) were merged into a single sequence file, run through the SEPP Fragment Insertion QIIME2 pipeline, and inserted into the Green Genes 13.8 tree. Unifrac distances based on the tree generated were used to run a Principal Coordinate Analysis (PCoA). PICRUSt2 was used to estimate gene functionality in the OTUs. The QIIME2-2021.2 version of the QIIME2 PICRUSt2 plug-in was utilized with EPA-NG sequence placement and “mp” hidden-state prediction. The data were analyzed using ALDEx2, which creates a centered log-ratio data distribution. T-tests with Benjamini-Hochberg correction were used for paired TP analyses.

**Table 1:** Fecal microbiota community indices for all rhesus macaque subjects at seven different TPs during the longitudinal study of P. *cynomolgi* infection.

Timepoints	Subjects (n)	OTUs	Chao1 indeces	Shannon	Faith’s PD
Baseline (1A)	6	6312	1066.01 ∓ 37.78	9.10 ∓ 0.27	32.08 ∓.98
Pre-peak (1B)	6	6264	1059.71 ∓ 28.88	9.01 ∓ 0.56	39.7 ∓ 5.51
Peak (2)	6	1962	334.37 ∓ 104.83	5.59 ∓ 0.42	26.08 ∓ 6.81
Post-peak (3)	6	5255	882.99 ∓ 168.67	8.66 ∓ 0.69	28.97 ∓ 2.18
Early relapse (3A)	5	4279	863.64 ∓ 210.3	8.57 ∓ 0.81	29.60 ∓ 2.24
Peak relapse (4)	4	4176	1060.32 ∓ 87.10	9.01 ∓ 0.54	33.78 ∓ 1.28
Relapse resolution (5)	5	4605	930.29 ∓ 109.64	9.00 ∓ 0.22	31.7 ∓ 1.96
Sub patent (7)	4	4874	1237.61 ∓ 42.85	9.63 ∓ 0.05	33.56 ∓ 0.66
	Total=42				

### 2.5 Whole metagenomics shotgun sequencing for species identification

To identify the species of bacteria from the significantly perturbed clades in the 16S analysis of our six-monkey infected cohort, we performed whole metagenomics shotgun sequencing on rectal swab DNA from four malaria-naive control rhesus. DNA from rectal swabs was isolated using the QIAGEN DNeasy PowerSoil Pro Kit, according to the manufacturer’s protocol. Extracted DNA samples were quantified using Qubit 4 fluorometer and Qubit™ dsDNA HS Assay Kit (Thermofisher Scientific). Between 10 and 20 million high quality reads were generated for each sample. Unassembled sequencing reads were directly analyzed by CosmosID-HUB Microbiome Platform (CosmosID Inc., Germantown, MD) described elsewhere (Hasan et al. (2014); Lax et al. (2014); Ottesen et al. (2016); Ponnusamy et al. (2016)) for multi-kingdom microbiome analysis and profiling of antibiotic resistance and virulence genes and quantification of microorganisms relative abundance. Briefly, the system utilizes curated genome databases and a high performance data-mining algorithm that rapidly disambiguates hundreds of millions of metagenomic sequence reads into the discrete microorganisms engendering the particular sequences. To identify the most abundant bacterial species in the gut microbiome of these control animals, we determined the relative abundance of all bacteria in the samples and calculated the relative abundance of each one in each sample. We then took the average relative abundance of each microbe, averaged across the four control animals, and ranked them in order of highest abundance. The top twenty most abundant bacterial species were determined to be the species most highly represented in the uninfected healthy rhesus macaque gut microbiome.

### 2.6 Untargeted metabolomics analysis of plasma samples

Plasma extraction and liquid chromatography mass spectrometry (LC-MS) analysis procedures were performed as described previously (Cordy et al. (2019)). Briefly, 50 *μ* l of plasma was spiked with 2.5 *μ* l of stable-isotope-labeled internal standards and 100 *μ* l of acetonitrile to precipitate protein. The clean extract was collected after centrifuging the plasma mixture at 14,000 g for 10 minutes at 4°C. 10 *μ* l was injected in triplicate into a Thermo Fisher Scientific Q Exactive HF high-field mass spectrometer using an autosampler maintained at 4°C. The sample order was randomized prior to LC-MS sample preparation, and then the samples were prepared for analysis in sequential batches. The metabolites were chromatographically separated using HILIC columns (Thermo Fisher Scientific Accucore 50 × 2.1 mm) with a 5-minute formic/acetonitrile gradient. Electrospray ionization was used in the positive-ion mode. Data quality was monitored by injecting 10 *μ* l of quality control samples (NIST SRM 1950 and internal standards) after every 20 samples. Raw data were preprocessed using apLCMS (Yu et al. (2009)) and xMSanalyzer (Uppal et al. (2013)) to extract retention time, *m/z*, and intensity information. Throughout the manuscript, an *m/z* feature refers to a unique combination of *m/z* and retention time. The preprocessed data were further treated to correct for batch effects using ComBat (Johnson et al. (2007)). The metabolic features were annotated and identified using xMSannotator (Uppal et al. (2013)). The untargeted LC-MS host plasma metabolomics data were analyzed using the R package xmsPANDA (Uppal (2021)). xmsPANDA output was then run through an annotation python script using MS/MS information from standardized metabolites that were analyzed on the same LC-MS machinery as the experimental samples. This annotation program had an error range of 30 seconds for retention time and 0.02 for mass to charge ratio. The median of three LC-MS replicates was used to quantify metabolite data. The LC-MS intensity data were used for plotting the values of kynurenine and tryptophan metabolites. In the longitudinal analysis shown in values below the limit of detection were represented as 0. For the ratio analysis of tryptophan and kynurenine, values under the limit of detection were analyzed as half of the minimum value seen at that TP. A paired student’s t-test was utilized for the ratio analysis.

## 3 Results

### 3.1 Host gut bacterial community structure during baseline, primary, and relapse malarial infection

**Figure 2:**
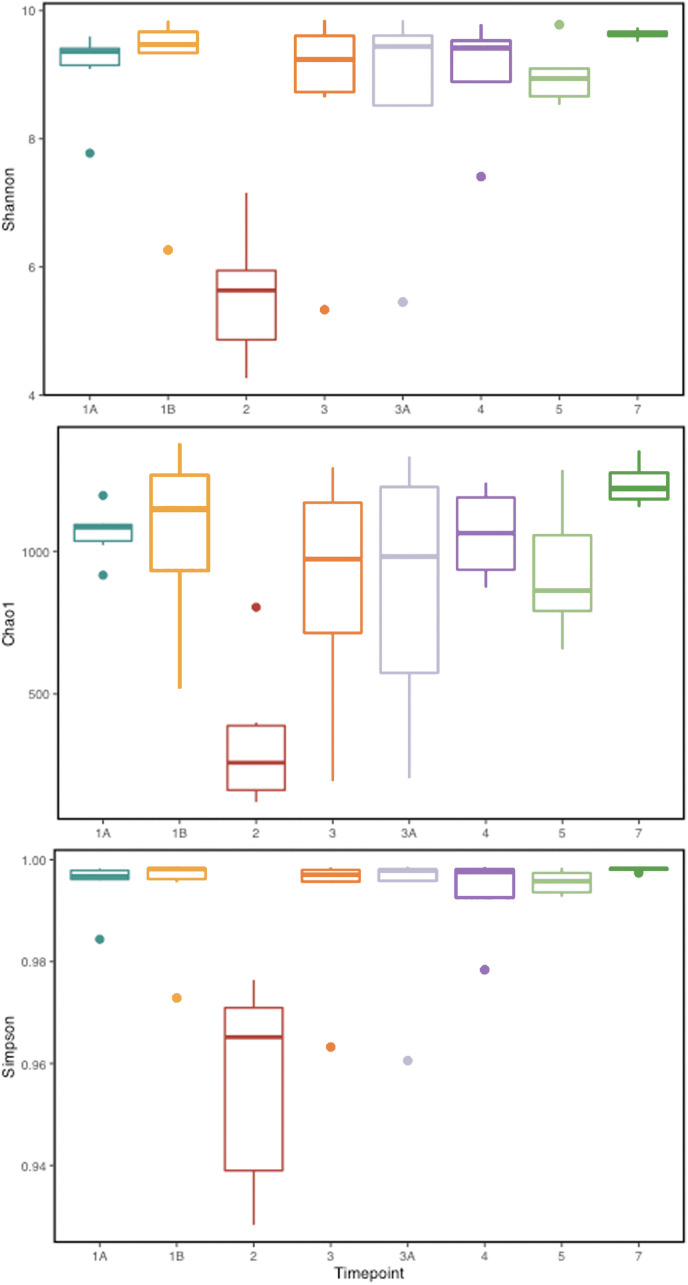
Alpha diversity metrics (Shannon, Simpson, and Chao 1 richness indices) for the gut microbial communities at various TPs during *P. cynomolgi* infection of rhesus macaques. All the panels point towards lowered microbial diversity, richness, and evenness during the peak *P. cynomolgi* infection TP.

To identify and catalog gut microbiota during malaria infections, we sequenced and analyzed 1,862,024 Illumina 16S rRNA reads originating from rectal swabs/feces samples from six na¨ıve rhesus macaques acquired at baseline and predefined TPs during a longitudinal P. *cynomolgi* infection study (Joyner et al., 2019). Sequence analysis at the phyla level showed that Firmicutes, Bacteroidetes, Proteobacteria and Spirochaetes were the four major phyla in all subjects at any given TP, accounting for >90% of the total bacterial communities in all samples (**Table S1**). Comparing all the TPs (**Figure 1A**), the difference between baseline (TP1A) and peak infection (TP2) was noteworthy. Proteobacteria increased dramatically during peak infection by TP2 while Firmicutes, Bacteroidetes, Spirochaetes amongst others decreased compared to baseline and other TPs as well (**Figure 1B**, **Table S1**). Members of the family *Helicobacteraceae*, which lies within the Epsilon subdivision of the Proteobacteria, were significantly greater in numbers during peak infection compared to the baseline microbial community structure (**Figure 1B**, depicted in blue). Members of *Ruminococcaceae* belonging to phylum Firmicutes, class Clostridia decreased considerably by TP2 (**Table S2**). Another notable family, *Prevotellaceae* belonging to the phylum Bacteroidetes, declined considerably by TP2 (**Figure 1B**, **Table S2**). ANOVA at the family level revealed *Helicobacteraceae* were significantly increased by TP2, as shown in **Figure 5**. Conversely, selected families such as *Streptococca ceae, Lactobacillaceae, Prevotellaceae*, and *Lachnospiraceae* were statistically lower in numbers during peak infection (**Figure 5**). Alpha diversity metrics support the above results, affirming lowered diversity and richness, as well as evenness during the peak phase of infection (**Table 1**; **Figure 2**).

### 3.2 Gut bacterial diversity in rhesus macaques

**Figure 3:**
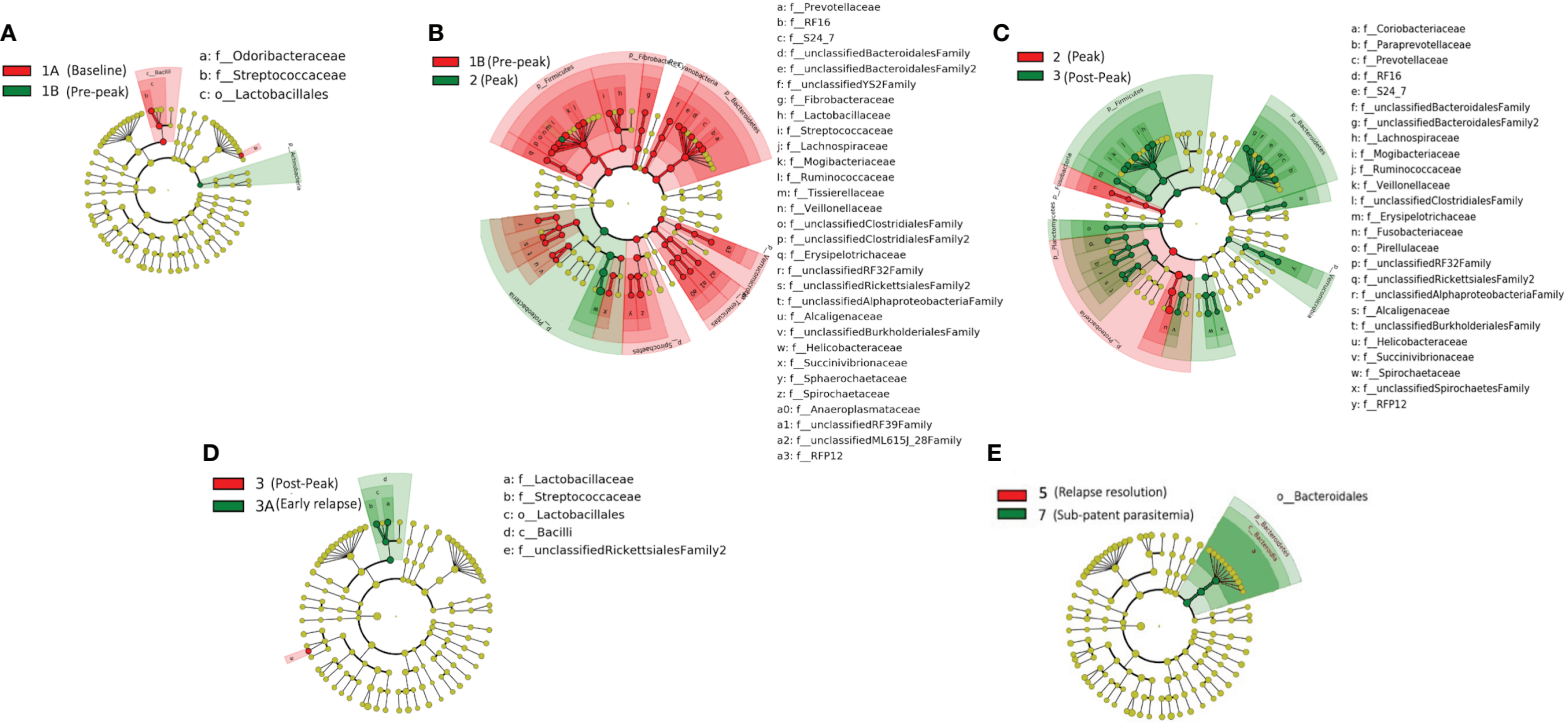
Taxonomic representation of statistically consistent differences between TPs during the longitudinal study of P. *cynomolgi* infection in rhesus macaques. LefSe cladograms from LDA analysis indicate significantly increased and decreased clades between TPs. **(A)** At the pre-peak stage (TP1B) *Lactobacillus* is decreased compared to baseline, and the phylum Actinobacteria is increased, although none of its contained clades were individually increased. **(B)** Between pre-peak (TP1B) and peak (TP2) 28 families decreased in abundance. Two families increased in abundance: *Helicobacterceae* and *Tissierellaceae*, both of which are anaerobic or aerotolerant. **(C)** Many taxa which decreased between pre-peak and peak infection (TPs 1B and 2) increased between peak and post-peak (TPs 2 and 3) indicating at least a partial restoration of the gut microbiome post-peak. **(D)** *Lactobacillus* continues to increase between post-peak and early relapse (TPs 3 and 3A). **(E)** While no significant changes were seen in the peak relapse (TP4), or relapse resolution (TP5), the bacteroidetes phylum is increased in the sub-patent samples compared to the relapse resolution (TPs 5 and 7). The insignificant data resulting from the TP3A/TP4 and TP4/TP5 comparisons is not shown in this figure.

We obtained a cumulative 37,727 OTU’s in all 42 samples from our six-monkey cohort, after clustering at a 97% similarity level (**Table 1**). A good sequencing depth for all samples was indicated by the rarefaction curve (subsampled at the lowest sample size) reaching asymptote as the number of sequences increased (**Figure S1**). The alpha diversity indices were calculated to evaluate the overall microbiota richness and diversity across the seven experimental TPs. The averages of Chao 1 richness indices for all subjects at each TP were calculated to estimate species richness in terms of the number of bacterial species present. To assess the evenness and relative abundance of the gut bacterial communities, we calculated the Shannon and Simpson diversity indices (Kim et al. (2017)). Based on the alpha diversity metrics, the overall fecal microbiota richness and diversity decreased considerably during peak infection at TP2 compared to baseline and relapse stages of infection (**Table 1**, **Figure 2**). This decrease suggests there is a loss of microbial diversity at peak malaria leading to host gut dysbiosis. To evaluate the similarity of gut microbial communities between the two most distinct TPs, 1A and 2, we examined the beta diversity using a PCoA plot based on weighted Unifrac distances as shown in **Figure S2**. The PCoA plot corroborated our previous results confirming that the gut microbiota at peak malarial infection was distinct from the baseline and relapse microbiota. The alteration in gut microbiome between the TPs in this study was analyzed using LEfSe (LDA score >2, p <0.05). The LEfSe results support the findings of increased *Helicobacteraceae* and decreased Firmicutes at TP2 (**Figure 3B**). This analysis also revealed increased *Lactobacillaceae* at TP3A (**Figure 3D**), the first relapse TP. *Lactobacillaceae* is often associated with gut health Turroni et al., 2014. Changes in taxa abundance were observed overwhelmingly in the primary infection compared to the relapse infection. No significant changes were detected between TPs 3A and 4 (Early relapse and peak relapse) nor TPs 4 and 5 (peak relapse and relapse resolution). Interestingly, of the 28 taxa that decreased between TP1B (pre-peak) and TP2 (peak) 19 of those increased between TP2 (peak) and TP3 (post-peak). These increases indicate a partial restoration of the gut microbiome post-peak (**Figure 3C**). The increases of *Helicobacteraceae*, an anaerobic, mucosal family, and the family *Tissierellaceae* (of which most members are anaerobic or aerotolerant), at peak infection. The increases of *Helicobacteraceae*, an anaerobic, mucosal family, and the family Tissierellaceae (of which most members are anaerobic or aerotolerant), at peak infection may indicate damage to the endothelium of the gut (**Figure 3B**). The results of this analysis indicated that the larger changes in mucosal versus luminal bacteria may be of interest.

We compared our results with previously published data (Yasuda et al. (2016)) on the biogeography of the intestinal mucosal and lumenal microbiome in rhesus macaques using PCoA plots. As shown in **Figure 6**, we found that the gut microbiota samples at TP2 clustered together with the reference mucosal microbiome samples from the previous study. However, the other TPs did not seem to have a notable correlation with either the mucosal or lumenal microbiome samples. These results suggest that, at peak malaria, there is an increase of mucosal derived taxa such as *Helicobacter* spp.

In addition, we analyzed the oxygen requirement profiles of the major bacterial clades at all TPs. The three predominant categories observed were the microaerophiles, obligate anaerobes, and facultative anaerobes (**Figure S3**). Interestingly, the relative percentage abundance of microaerophiles increases at TP2 (Solnick and Vandamme (2001); Gueneau and Loiseauxdegoer (2002)), coinciding with the peak levels of P. *cynomolgi*, which is also a microaerophile.

### 3.3 Identification of microbial species using metagenomic sequencing

To determine the identities of the most abundant bacterial species perturbed in the gut microbiome, we performed whole metagenome sequencing on rectal swab samples collected from four control rhesus macaques. The bacterial species found in highest abundance in the *Helicobacteraceae* family was *Helicobacter macacae*, a known commensal microbe of rhesus macaques, previously identified in Yasuda et al. and other reports, but typically shown to be adherent to the mucosa and not present in high numbers in the luminal contents (Yasuda et al. (2016)). Additional bacterial species identified included members of the *Prevotellaceae* family (Prevotella copri) and the *Lactobacillaceae* family (*Lactobacillus johnsonii,Ligilactobacillus animalis, and Limosilactobacillus reuteri*) (**Figure S4**).

### 3.4 Host metabolic analysis

**Figure 4:**
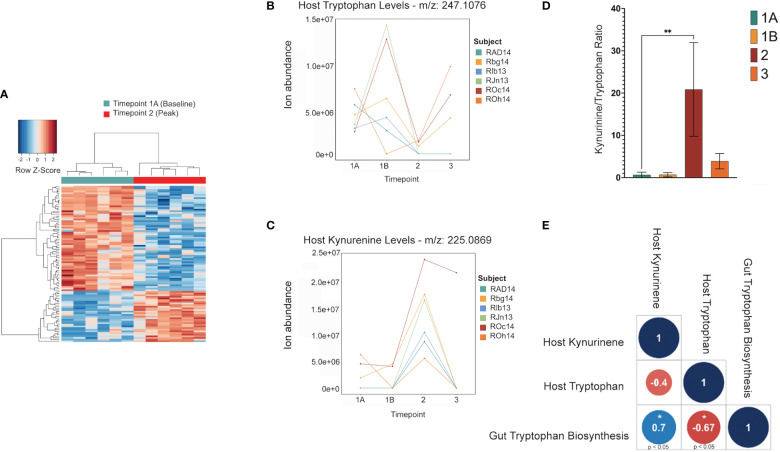
Shifts in the host tryptophan/kynurenine pathway may be linked to microbial production of tryptophan in the gut. This data shows major shifts in the host metabolome where red indicates a significant increase, and blue indicates a significant decrease **(A)**. LC-MS data of host metabolite samples throughout primary infection (TP1A-TP3, n=9) reveals Kynurinine peaks a TP2, while tryptophan decreases at TP2. Data shown is separated and colored by subject for trend clarity. This shift in the kynurenine/tryptophan ratio indicates IDO activity **(B, C)**. The tryptophan/kynurenine ratio across the primary infection (TP1A-TP3, n=9) shows a significant shift (p=0.0073) from tryptophan to kynurenine in the host at TP2 **(D)**. Spearman correlation shows host kynurenine levels and host tryptophan levels to be linked to gut microbiome tryptophan production across the primary infection (p=0.0358, p=0.0499) **(E)**.

Untargeted metabolomics was performed on plasma collected from venous blood taken from the same animals at the same TPs in which rectal swabs were collected. Using LC-MS and a high resolution metabolomics workflow (Cordy et al. (2019)), dynamic shifts in multiple metabolites were shown to occur during the peak infection, at TP2. Between TP1A and TP2, 95 metabolites were found to be significantly changed in abundance (**Figure 4A**). Using previously published methodology involving the comparison of mass:charge *(m/z)* ratios and retention times for reference compounds run on the same machine, we were able to annotate seven of them (**Table 2**). Among the seven, two members of the kynurenine pathway, acetyltryptophan and hydroxykynurenine, were identified. Acetyltryptophan peaked at TP1B and dropped significantly at TP2 while hydroxykynurenine peaked at TP2 and dropped significantly at TP3A (**Figures 4B–D**; **Table 2**). Other metabolic changes measured included a decline in hypoxanthine and an increase in multiple fatty acyl carnitines during peak parasitemia.

**Figure 5:**
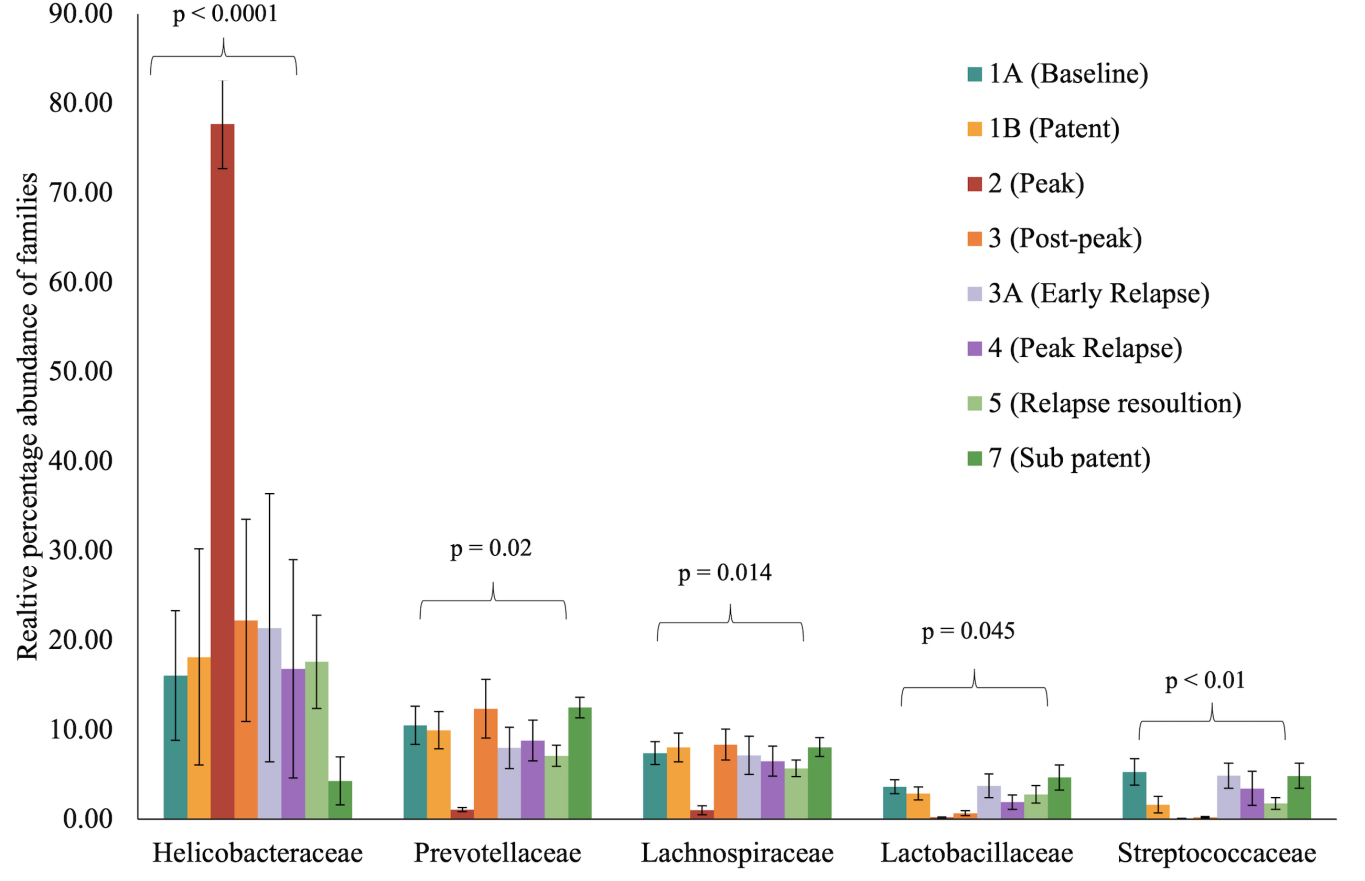
Selected bacterial families with significant variance during malarial infection in rhesus macaques study model. The data (mean ± SE) for relative percentage sequence abundance of each family at each timepoint were compared using one-way analysis of variance repeated measures (ANOVA) test (p ≤ 0.05). P-value denotes the result of the ANOVA comparing all TPs for one family.

**Figure 6:**
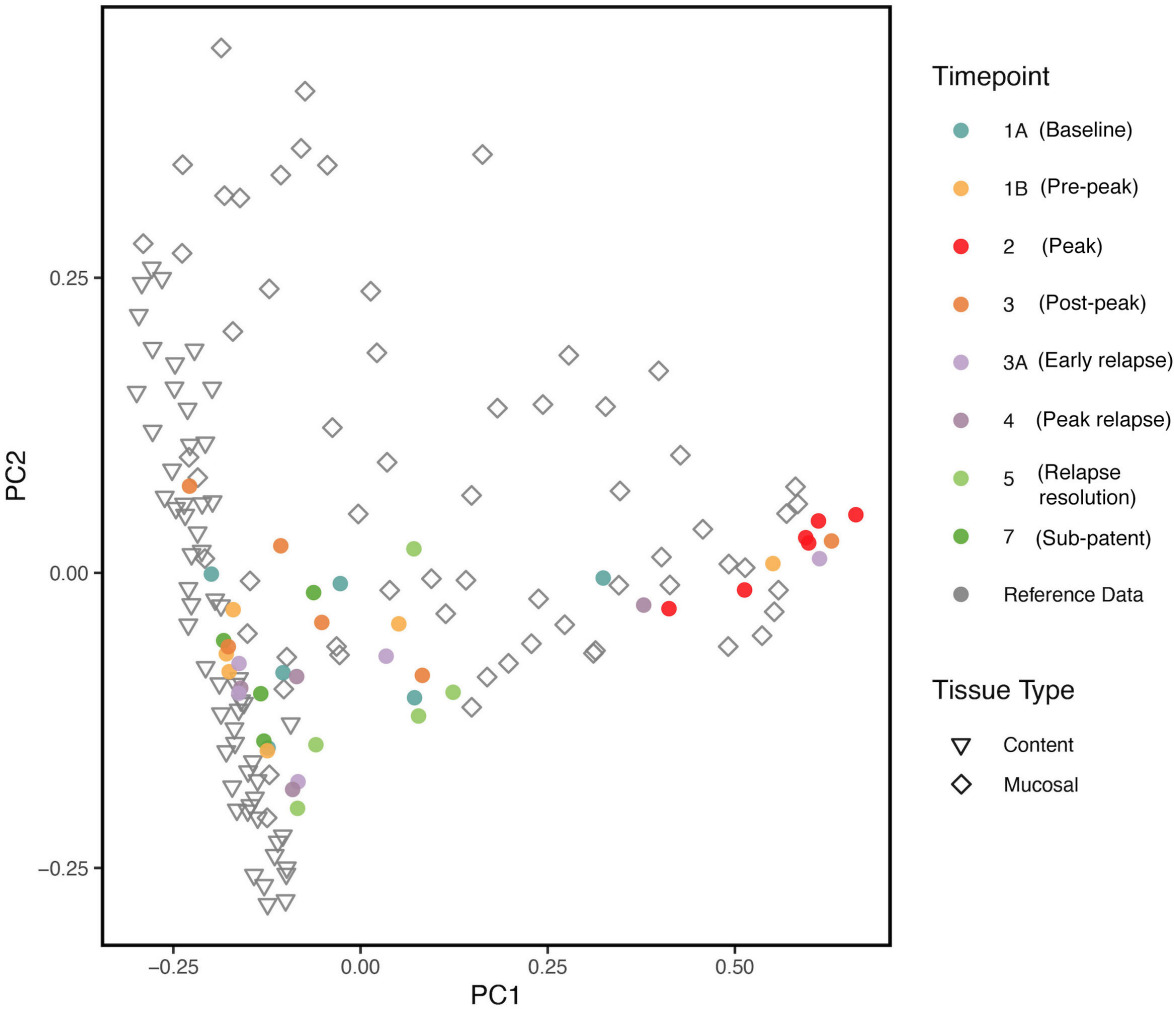
PCoA plot comparing, at various TPs, the gut microbial communities from the longitudinal P. *cynomolgi* infections, with previously published data on the lumen and mucosal bacterial diversity in rhesus macaques. Each marker represents one sample, and the colors are depictive of the different TPs. PCoA indicates that microbiome samples from peak infection (TP2) are more similar in microbial composition to healthy mucosal samples than healthy luminal samples.

**Table 2:** Mass-to-charge ratio and retention time input values of significant host metabolites as found by xmsPANDA.

Input *m/z*	Input retention time	Fold change	Possible identities
260.1854	64.1	-2.563738333	Hexanoylcarnitine
247.1076	32.5	2.478281667	Acetyltryptophan
194.0812	31.5	-3.268476667	2-Methylhippurate [3-Methylhippurate, 4-Methylhippurate, O-Methylhippurate, Methylhippurate]
137.0458	63.3	3.446455	Hypoxanthine
225.0869	58.2	-2.717815	Hydroxykynurenine
400.3415	61.3	-3.57693	Palmitoylcarnitine
288.2168	61.3	-2.595535	Octanoylcarnitine

Possible identities indicated if within 0.02 m/z and 30 seconds of retention time. Isomers shown in brackets. Fold change reflects the difference between TP1 and TP2.

### 3.5 Functional capability analysis

A software program for predicting the functions of microbes within a microbial community based on 16S data alone was used to assess whether microbes could have any involvement in the metabolic changes occurring in the host. Phylogenetic Investigation of Communities by Reconstruction of Unobserved States (PICRUSt2) (Douglas et al. (2020)) was used to estimate active metabolic pathways of the gut microbiome at each TP. This revealed 124 significant MetaCyc pathways between TP1A and TP2 (t-test, p ≤ 0.05), and 592 significant individual predicted enzymes classified by Enzyme Commission number (EC) between TP1A and TP2. ECs connected to tryptophan metabolism as described previously (Kaur et al. (2019)) were specifically analyzed to investigate ties to the host metabolic shift seen at TP2, but all enzymes were either not present in our data or were insignificant. However, the pathway analysis did reveal a significant increase in the L-tryptophan biosynthesis pathways at TP2 (MetaCyc ID : TRPSYN-PWY) and its superpathway (MetaCyc ID: COMPLETE-ARO-PWY), suggesting the possibility of increased capacity for producing tryptophan by the gut microbiota of the macaques while experiencing peak parasitemia. A Spearman correlation was used to evaluate a possible association between the prevalence of the tryptophan biosynthesis pathway in the gut and host tryptophan and kynurenine levels during primary infection (TP1A-TP3). This analysis revealed host kynurenine and tryptophan levels to be highly correlated with gut tryptophan production, although not significantly correlated with each other (**Figure 4E**).

## 4 Discussion

Studies attempting to understand the possible impact of *Plasmodium* infection on the mammalian host microbiota over the course of malaria are limited (Mukherjee et al., 2020). This study, based on the P. *cynomolgi*-rhesus macaque infection model, focuses on expanding this horizon. We present a systematic longitudinal study to assess whether P. *cynomolgi* infection affects the composition of the microbiota in rhesus macaques over a period of time. At the phyla level, the relative percentage abundance of Proteobacteria was thrice as high at peak parasitemia (TP2) compared to the baseline (TP1A), as shown in **Table S1**. This phylum contains several pathogenic and opportunistic pathogenic microorganisms. Within the Proteobacteria, there was an increase in the number of *Helicobacteraceae* family members at peak infection (**Table S2**), accounting for nearly 90% of the gut microbiota at TP2. Using shotgun sequencing applied to another set of rhesus macaques from the same animal facility (not malaria-infected), we were able to confirm the presence of bacteria of the *Helicobacter* genus in these animals, and the species was confirmed to be *Helicobacter macacae*. Members of *Helicobacter* spp. are predominantly microaerophiles, requiring environments containing lower levels of oxygen than are present in the atmosphere and have been associated with gastric colonization (Dewhirst et al. (2000); Iten et al. (2001); Gueneau and Loiseauxdegoer (2002)). Interestingly, *Plasmodium* is also a microaerophile, growing most rapidly under low oxygen conditions. While oxygen wasn’t directly measured in this study, this finding does point to the possibility of an overall metabolic change in the host’s environment that may be selective for increased survival and replication of microaerophiles such as *Plasmodium* and *H. macacae*.

At TP2, the number of members of the bacterial families *Ruminococcaceae* (phylum Firmicutes) and *Prevotellaceae* (phylum Bacteroidetes) declined. Both *Ruminococcaceae* and *Prevotellaceae* have been previously associated with gut health (Rinninella et al. (2019)). Linear Discriminate Analysis of our data revealed an increase in *Lactobacillus* at TP3A, the first TP in the relapse phase of infection. *Lactobacillus* is often correlated with gut health, and this increase may indicate a period of gut health between the primary and relapse stages. The increased prevalence of *Helicobacteraceae* and *Tissierellaceae* bacteria are suggestive that either the gut luminal environment became more hospitable for mucosal microbes, or that mucosal microbes were displaced from the endothelial mucosa. This gut dysbiosis at peak parasitemia could possibly be altering the metabolic processes regulated by a balanced intestinal microbiome.

Comparing our experimental TP dataset with previously published data (Yasuda et al. (2016)), it was found that, at peak malaria TP2, the gut microbiome samples clustered closely with the reference mucosal microbiome of healthy rhesus macaques. This clustering may be suggestive of the “sloughing off” effect, where *Plasmodium* infection damages the gut endothelium, displacing mucosal tissue and its associated microbiota. This “sloughing off” effect could also indicate membrane weakness, which would allow more exchange in metabolites between the gut and the host bloodstream. Thus, it is critical to consider both the functional pathways of the gut microbiome and the host metabolome when assessing how host infection impacts the gut microbiome.

Functional analysis of the gut microbes revealed a significant increase in the genetic capacity for tryptophan synthesis at peak infection (TP2) when *H. macacae* is elevated, which coincides with the increase in kynurenine production from tryptophan in the host during the primary stage of infection spanning TP1A through TP3 (**Figures 4B–E**). As the bacterial tryptophan synthesis pathway is negatively regulated by tryptophan, an intriguing possibility is that as the host uses up tryptophan to produce kynurenine, the gut microbiota produces more tryptophan that feeds into the pathway. This could explain why, in previous studies of the plasma metabolome in acute malaria, kynurenine was significantly enriched while tryptophan was not significantly depleted (Cordy et al. (2019)). Evidence from *Helicobacter pylori* mucosal infections also supports this hypothesis. Multiple studies have found that expression of IDO is enhanced in subjects with *H. pylori* mucosal infections (Azadegan-Dehkordi et al. (2021)). This finding suggests that the IDO upregulation detected in our data may be partly due to the increase in *Helicobacteraceae* at peak parasitemia, perhaps related to the ability of *H. macacae* to produce tryptophan, which is converted to kynurenine by IDO. While it is unclear what environmental pressures lead to the increase in *Helicobacter* in the rectal swabs during TP2, and thus this correlation with bloodstream kynurenine pathway activity may be coincidence, it is certainly an intriguing hypothesis worthy of future follow-up research. Overall, these results support that tryptophan is a key metabolite produced during malarial infection, and, in particular, is related to the *Plasmodium*-host-microbiota tripartite relationship. Furthermore, the shifts in microbiota detected at peak parasitemia indicate that the gut microbiome is most affected during peak primary parasitemia, and, to a lesser extent, during subsequent relapse parasitemias. This finding suggests that the gut microbiome is more greatly impacted by the acute systemic metabolic and immune responses of the host than by the mere presence of parasites.

## Data availability statement

The 16S data presented in the study are deposited in the Sequence Reads Archive (SRA), accession number PRJNA907388. The shotgun sequencing data presented in the study are deposited in the Sequence Reads Archive (SRA), accession number PRJNA907397. The metabolomics data presented in the study are deposited on the MetaboLights repository, accession number MTBLS54265.

## Ethics statement

The animal study was reviewed and approved by Institutional Animal Care and Use Committee (IACUC) at Emory University.

## Author contributions

Conceived and designed the experiments: MC, CJ, SL, AM, MG, RC, and members of the MaHPIC-Consortium. Performed the experiments: VT, MC, CJ, SL, and RC. Performed data analysis: DF, SK, and RC. Interpreted the data analysis: DF, SK, and RC. Managed and led validation and quality control of datasets and deposited the data and metadata: VT, SP, MN, JD, JK, and RC. Generated the figures: DF, SK, and RC. Wrote the paper: DF, SK, and RC. Provided manuscript editorial contributions: MC, CJ, JD, JK, SL, AM, and MG. All authors read and approved the final manuscript.
