# Supplementary material for: Malaria disrupts the rhesus macaque gut microbiome
Source: Front Cell Infect Microbiol. 2023 Jan 13;12:1058926. doi: 10.3389/fcimb.2022.1058926 (PMC9880479; doi:10.3389/fcimb.2022.1058926)
Supplement: Supplementary file 1 [file DataSheet_1.pdf]

Supplementary Material

1 SUPPLEMENTARY TABLES AND FIGURES

1.1 Tables

Table S1. Relative percentage abundance of predominant phyla. Phyla listed comprised of at least 0.1% of reads from at least one sample. Abundances of 16S reads were first normalized to proportional abundances and then the average proportional abundance (n=6,5/4) for each phylum was calculated for rhesus macaques from each of the timepoints.

	Baseline	Pre-peak	Peak	Post-peak Relapse	Early Relapse	Peak resolution	Relapse	Sub patent
	1A	1B	2	3	3A	4	5	7
Firmicutes	47.47	43.48	11.51	39.74	46.08	44.58	41.87	49.25
Bacteroidetes	21.59	22.39	4.75	23.41	18.71	20.19	19.97	25.44
Proteobacteria	20.84	22.32	78.75	26.21	24.63	21.16	21.86	9.68
Spirochaetes	4.52	6.06	3.23	6.57	5.51	6.86	8.17	8.88
Fibrobacteres	1.23	0.64	0.06	0.40	0.85	1.39	1.00	0.36
Tenericutes	0.88	1.05	0.18	0.89	0.89	1.36	1.53	1.39
Cyanobacteria	1.22	1.19	0.13	0.69	0.87	1.23	1.57	1.45
Unassigned Bacteria	0.52	0.59	0.32	0.21	0.35	0.61	0.75	0.64
Lentisphaerae	0.46	0.46	0.17	0.35	0.44	0.93	1.15	0.70
Verrucomicrobia	0.77	0.80	0.13	0.77	0.73	1.06	1.27	1.38
WPS-2	0.21	0.49	0.14	0.15	0.37	0.23	0.28	0.45
Elusimicrobia	0.07	0.10	0.04	0.06	0.08	0.05	0.22	0.10
Actinobacteria	0.10	0.29	0.36	0.43	0.39	0.31	0.22	0.14

Table S2. Table S2. Relative percentage abundance of predominant families Families listed comprised of at least 0.1% of reads from at least one sample. Abundances of 16S reads were first normalized to proportional abundances and then the average proportional abundance (n=6,5/4) for each family was calculated for rhesus macaques from each of the timepoints.

		Baseline 1A	Pre-peak 1B	Peak Relapse 2	Post-peak Relapse 3	Early resolution 3A	Peak 4	Relapse 5	Sub patent 7
Proteobacteria	Helicobacteraceae	16.09	18.15	77.69	22.26	21.41	16.85	17.61	4.30
	Alcaligenaceae	0.93	0.70	0.12	0.71	0.43	0.87	0.78	1.07
	Succinivibrionaceae	0.46	0.62	0.06	0.55	1.15	0.59	0.70	0.58
Bacteroidetes	Prevotellaceae	10.53	9.98	1.10	12.39	8.00	8.83	7.11	12.50
	[Paraprevotellaceae]	2.17	2.28	0.93	2.73	2.12	2.22	2.52	3.31
	Bacteroidales_unassigned	3.51	4.23	0.88	3.03	3.59	3.36	4.66	3.55
	BacteroidalesRF16	1.61	1.90	0.40	1.52	1.58	1.85	2.15	1.94
	Bacteroidaceae	0.13	0.11	0.36	0.21	0.12	0.10	0.12	0.07
	Porphyromonadaceae	0.60	0.61	0.33	0.53	0.42	0.50	0.46	1.06
	Bacteroidales_p-2534-18B5	0.22	0.48	0.28	0.27	0.59	0.84	0.49	0.58
Firmicutes	Ruminococcaceae	17.23	16.97	5.18	15.94	17.34	17.14	17.22	17.42
	[Tissierellaceae]	0.09	0.23	1.21	0.52	0.39	0.57	0.42	0.02
	Lachnospiraceae	7.42	8.06	1.03	8.37	7.17	6.51	5.70	8.08
	Clostridiales_unassigned	4.57	4.29	0.99	3.32	3.70	4.67	4.69	4.77
	Christensenellaceae	1.02	1.41	0.64	1.41	1.47	1.35	1.37	1.27
	Erysipelotrichaceae	1.88	1.97	0.51	2.43	1.42	1.79	1.64	2.03
	Clostridiaceae	0.84	0.93	0.42	2.36	1.10	1.51	1.00	1.10
	Veillonellaceae	2.46	1.68	0.35	2.09	1.55	2.09	1.79	2.33
	Clostridiales_2unassigned	2.08	2.00	0.32	1.22	1.84	2.29	2.51	1.71
	[Mogibacteriaceae]	0.66	0.85	0.26	0.77	0.91	0.73	0.65	0.69
	Lactobacillaceae	3.66	2.91	0.23	0.71	3.76	1.93	2.82	4.68
	Streptococcaceae	5.30	1.67	0.10	0.25	4.90	3.48	1.77	4.87
	Peptococcaceae	0.11	0.12	0.06	0.10	0.15	0.16	0.09	0.13
Spirochaetes	Brachyspiraceae	0.53	0.62	2.33	2.48	1.35	0.93	0.78	0.16
	Spirochaetaceae	3.61	4.99	0.81	3.62	3.81	5.54	6.79	8.20
	Sphaerochaetaceae	0.35	0.39	0.09	0.41	0.29	0.31	0.49	0.48
Unassigned	Unassigned	0.52	0.59	0.32	0.21	0.35	0.61	0.75	0.64

1.2 Figures

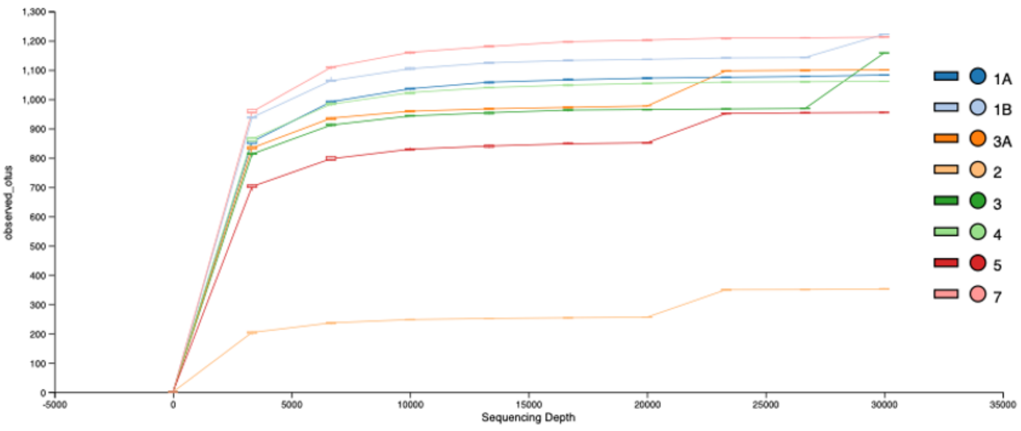

Figure S1. Rarefaction plot for the gut microbial communities at various TPs showing curves reaching asymptote at the cutoff of 30,000 reads. This rarefaction plot shows that the number of OTUs represented does not increase when analyzing 30000 or more sequences. This indicates that analyzing 30000 reads per sample will generate representative data.

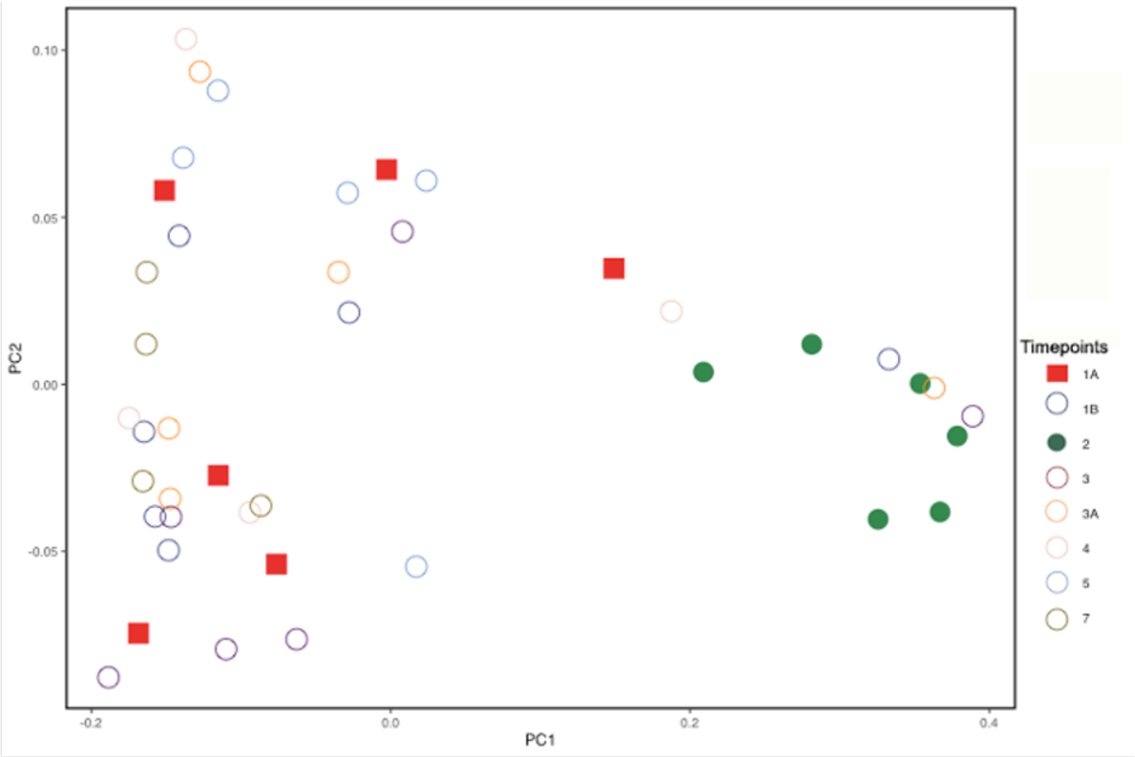

Figure S2. Peak parasitemia samples cluster separately from baseline in analysis of bacteria diversity. PCoA of bacterial diversity based on weighted UniFrac distances reveal that the fecal microbiota from the TP2 cluster distinctly from the baseline (TP1A) and other TPs in the longitudinal scale of this *P. cynomolgi* infection study. Each marker represents one sample, and the colors are depictive of the different TPs.

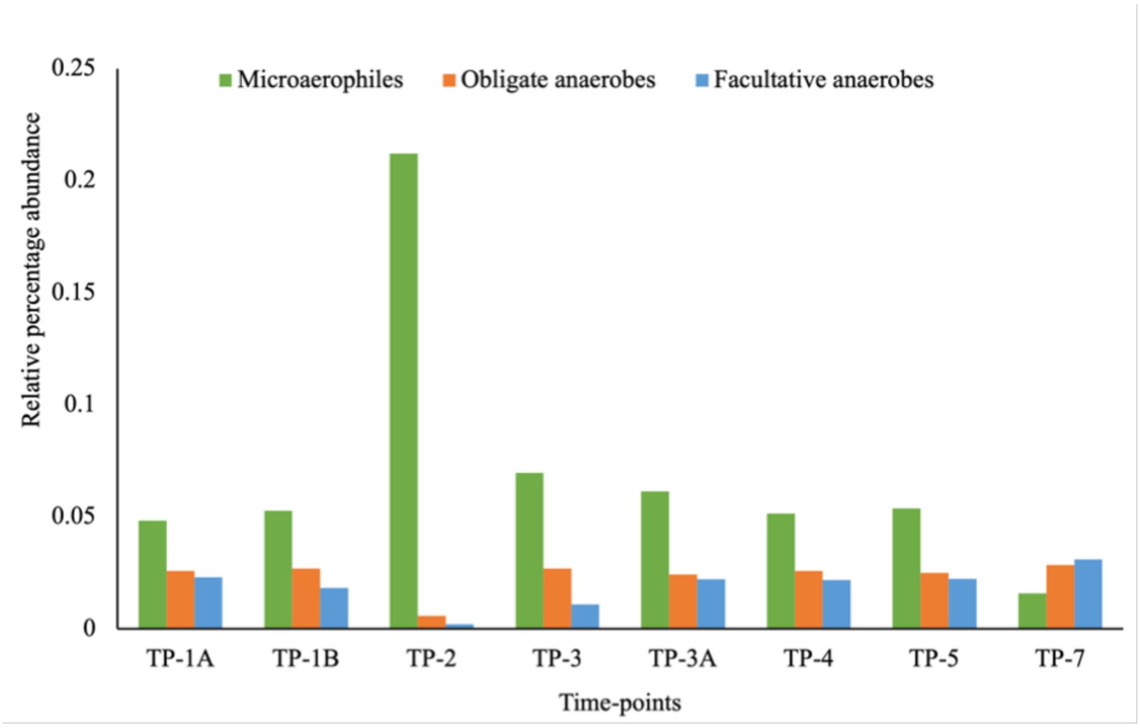

Figure S3. Microaerophiles are significantly increased at peak parasitemia compared to baseline. Oxygen requirements in correlation with predominant genera greater than 1% relative percentage abundance at all TPs during the longitudinal study of *P. cynomolgi* infection in rhesus macaques.

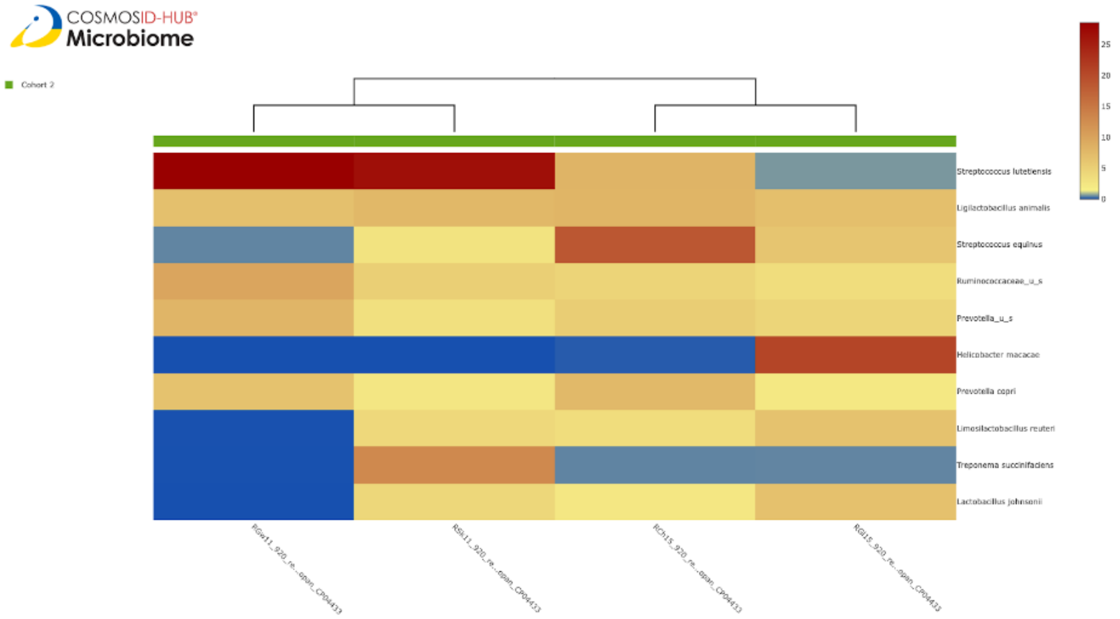

Figure S4. Whole metagenome sequencing of healthy macaques. Whole metagenome sequencing was performed on rectal swabs of four non-malaria naive, non-infected rhesus macaques who had recently been treated to clear parasites. The most abundant *Helicobacteraceae* family member in healthy macaques was found to be *Helicobacter macacae*.
